# Lobetyolin ameliorates DSS-induced ulcerative colitis in mice by alleviating inflammation, restoring barrier function, and modulating gut microbiota–metabolite interactions

**DOI:** 10.3389/fmicb.2025.1710707

**Published:** 2025-11-28

**Authors:** Min Chang, Fengping Liu, Junhui Lu, Qing Wang, Jingwei Liu, Xing Chen

**Affiliations:** 1 The First Clinical Medical College, Shanxi Medical University, Taiyuan, China; 2 Department of Gastroenterology, Heping Hospital Affiliated to Changzhi Medical College, Changzhi, China; 3 Department of Gastroenterology, The First Hospital of Shanxi Medical University, Taiyuan, China

**Keywords:** lobetyolin, ulcerative colitis, inflammation, gut microbiota, short-chain fatty acids, metabolomics

## Abstract

Ulcerative colitis (UC) is a chronic inflammatory bowel disease characterized by persistent mucosal inflammation in the colon, leading to substantial morbidity. Current therapies are often limited by side effects and relapse, emphasizing the need for safer, multi-target alternatives. This study investigated the protective effects and underlying mechanisms of lobetyolin (LBT), a natural polyacetylene glycoside, in a dextran sulfate sodium (DSS)-induced colitis mouse model. Male BALB/c mice were randomly divided into four groups: Control, DSS, and DSS treated with low (10 mg/kg) or high (50 mg/kg) doses of LBT. Clinical parameters were assessed using the disease activity index (DAI), histopathological staining, and biochemical assays. Inflammatory and oxidative stress markers were quantified by ELISA, tight junction proteins were analyzed by Western blotting and immunohistochemistry, gut microbiota composition was determined by 16S rRNA sequencing, and short-chain fatty acids (SCFAs) were measured by GC–MS. In addition, non-targeted metabolomics was performed using UHPLC–MS/MS. LBT treatment significantly alleviated DSS-induced colitis by improving body weight, colon length, and histological structure. It reduced TNF-*α*, IL-6, and IL-1β levels, restored antioxidant capacity (SOD, CAT, GSH), and enhanced epithelial barrier integrity (Occludin, Claudin-1, ZO-1). Moreover, LBT normalized gut microbial composition, increased SCFA production, and regulated amino sugar and nucleotide sugar metabolism. Collectively, these findings demonstrate that LBT exerts multi-target protective effects against UC by modulating inflammation, oxidative stress, epithelial barrier function, gut microbiota, and metabolic pathways.

## 1 Introduction

Ulcerative colitis (UC) is a chronic, relapsing inflammatory bowel disease (IBD) characterized by continuous mucosal inflammation of the colon and rectum (Kucharzik et al., 2020). The prevalence of fecal incontinence in patients with UC, even in remission, significantly increases medical and social costs and triggers anxiety, depression, and social isolation, constituting a serious but often overlooked public health hazard (Vasant et al., 2023). Although the exact etiology of UC has not been fully elucidated, available studies strongly suggest that its pathogenesis is the result of a multidimensional interaction between a genetic predisposition background, an imbalance in mucosal immune response, intestinal microecological disorders, defects in the epithelial barrier function, metabolic abnormalities, and environmental factors, such as diet, smoking, and infections (Świrkosz et al., 2023). UC is mainly manifested as diarrhea with blood, abdominal pain, and often accompanied by arthralgia, skin rashes and other extraintestinal symptoms, repeated episodes can lead to anemia, malnutrition, and even the need for surgical removal of the colon (Pantic et al., 2022). Current drug therapies such as aminosalicylates, corticosteroids, immunosuppressants, and biologics can reduce symptoms and induce remission, but suffer from the inability to directly correct dysbiosis as well as the fact that prolonged use of these medications can lead to high rates of relapse, adverse effects, and escalating treatment costs (Pai et al., 2021). Therefore, it is imperative to explore novel therapeutic strategies with better safety profiles and multi-target efficacy.

Growing attention has been directed toward the role of gut microbiota and their metabolites in the development and progression of UC (Cheng et al., 2023). In healthy individuals, the gut microbiota maintains intestinal homeostasis by regulating immune responses, supporting barrier function, and producing beneficial metabolites such as short-chain fatty acids (SCFAs) (Kim, 2023). In UC, microbiota dysbiosis is characterized by reduced bacterial diversity, decreased abundance of beneficial taxa, and increased prevalence of pathogenic bacteria (Pisani et al., 2022). These alterations are closely associated with compromised epithelial barrier integrity, overproduction of pro-inflammatory cytokines, and changes in metabolic profiles (Deleu et al., 2023; Poppe et al., 2024). Among microbial metabolites, SCFAs-particularly acetate, propionate, and butyrate-play essential roles in modulating mucosal immunity, suppressing inflammatory signaling, and maintaining epithelial health (Ney et al., 2023). Restoring gut microbial balance and enhancing SCFA production have thus emerged as promising strategies for UC prevention and treatment.

Another critical pathological feature of UC is the disruption of the intestinal epithelial barrier (Quansah et al., 2023). Tight junction proteins such as Occludin, Claudin1, and ZO-1 are vital for maintaining barrier integrity, while goblet cells and their mucin production form an essential protective layer against luminal antigens (Zhang, 2025). Oxidative stress further exacerbates epithelial injury, amplifies inflammatory cascades, and perpetuates tissue damage (Wang et al., 2023). Therefore, an effective therapeutic intervention should ideally target multiple pathological processes, including inflammation, oxidative stress, barrier dysfunction, microbiota dysbiosis, and metabolic imbalance.

Natural bioactive compounds have attracted considerable attention as multi-target agents for inflammatory bowel disease (IBD) therapy due to their broad pharmacological activities and favorable safety profiles (Xu et al., 2022). Lobetyolin (LBT), a polyacetylene glycoside isolated from *Codonopsis pilosula*, has been reported to possess anti-inflammatory, antioxidant, and immunomodulatory properties, suggesting potential therapeutic value in inflammation-related disorders (Chen et al., 2024). Previous studies have shown that LBT can suppress pro-inflammatory cytokines (e.g., TNF-*α*, IL-6, IL-1β) and enhance antioxidant defenses (SOD, CAT, and GSH) to alleviate oxidative stress (Huang et al., 2025a; Xiu et al., 2025). Therefore, it is hypothesized that LBT may ameliorate UC by suppressing inflammation, restoring epithelial barrier integrity, and regulating gut microbiota-metabolite interactions.

Nevertheless, while probiotics and various plant-based compounds have shown promise in modulating gut microbiota and strengthening epithelial barrier function, the ability of a single bioactive molecule to exert comparable multi-target effects remains largely unexplored. In particular, the interactions between LBT, gut microbial composition, and microbial metabolites in the context of UC have not yet been systematically investigated. Building on previous reports of the anti-inflammatory and antioxidant potential of LBT, this study aimed to address this critical gap by evaluating its protective effects in dextran sulfate sodium (DSS)-induced colitis in mice. We further sought to compare its regulatory actions with other microbiota-targeted strategies, such as probiotics or plant-derived polyphenols, to highlight the novelty of LBT as a natural compound capable of simultaneously modulating microbial balance, redox homeostasis, and epithelial integrity. Collectively, our findings provide new insights into the mechanisms through which LBT mitigates UC and support its development as a promising microbiota-associated therapeutic candidate.

## 2 Materials and methods

### 2.1 Materials and reagents

Six-week-old specific-pathogen-free (SPF) male BALB/c mice (8 mice per group) were purchased from SiPeiFu Biotechnology Co. (Beijing, China). The animals were housed in a clean, well-ventilated facility under controlled conditions with a 12 h light/dark cycle, an ambient temperature of 20–25 °C, and relative humidity of 50–60%. All mice were allowed to acclimatize to the environment for 1 week before the experiments. The experimental procedures were approved by the Animal Protection and Utilization Committee of Changzhi Medical College, Shanxi, China, and were conducted in strict accordance with the ethical guidelines of the World Organization for Animal Health. Standard rodent chow (Co60 radiation-sterilized) was obtained from Open Source Animal Feed (Changzhou) Co. LBT (purity ≥ 98.0%) was purchased from Shanghai Jinpan Biotechnology Co., Ltd. In general, LBT is extracted from the dried roots of *Codonopsis pilosula* by ethanol extraction, followed by chromatographic purification such as column chromatography or HPLC, and final crystallization. Its identity and purity are verified by spectroscopic methods including UV, IR, NMR, and MS analysis. Chemically, LBT is a polyacetylene glycoside characterized by a terminal alkyne group and a glycosidic linkage, which can undergo addition or oxidation reactions and hydrolysis under acidic conditions. DSS (purity ≥ 99.0%) was purchased from Yeasen Biotechnology (Shanghai) Co., Ltd. hematoxylin and eosin (H&E), Alcian Blue Periodic Acid Schiff (AB-PAS) Stain Kit and immunohistochemistry kit were purchased from Beijing Solarbio Biotechnology Co. Ltd. Superoxide dismutase (SOD), glutathione (GSH), catalase (CAT), malondialdehyde (MDA), tumor necrosis factor *α* (TNF-α), interleukin 6 (IL-6), interleukin 1*β* (IL-1β), lipopolysaccharide (LPS), mouse mucin 2 (MUC2), and myeloperoxidase (MPO) test kits were purchased from Xiamen Lunchangshuo Bio-technology Co. Antibody of rabbit anti β-actin (l102) was purchased from Bioworld Technology, Inc. (Nanjing, China). Antibodies of rabbit anti Occludin (A2601), rabbit anti Claudin 1 (A2196) and rabbit anti ZO-1 (A 11417) were purchased from ABclonal Technology Co., Ltd. (Wuhan, China). HRP Anti-Rabbit IgG (BA1054) was purchased from Wuhan Boster Biological Technology Co. Ltd (Wuhan, China).

### 2.2 Animal experiment design

**Figure 1:**
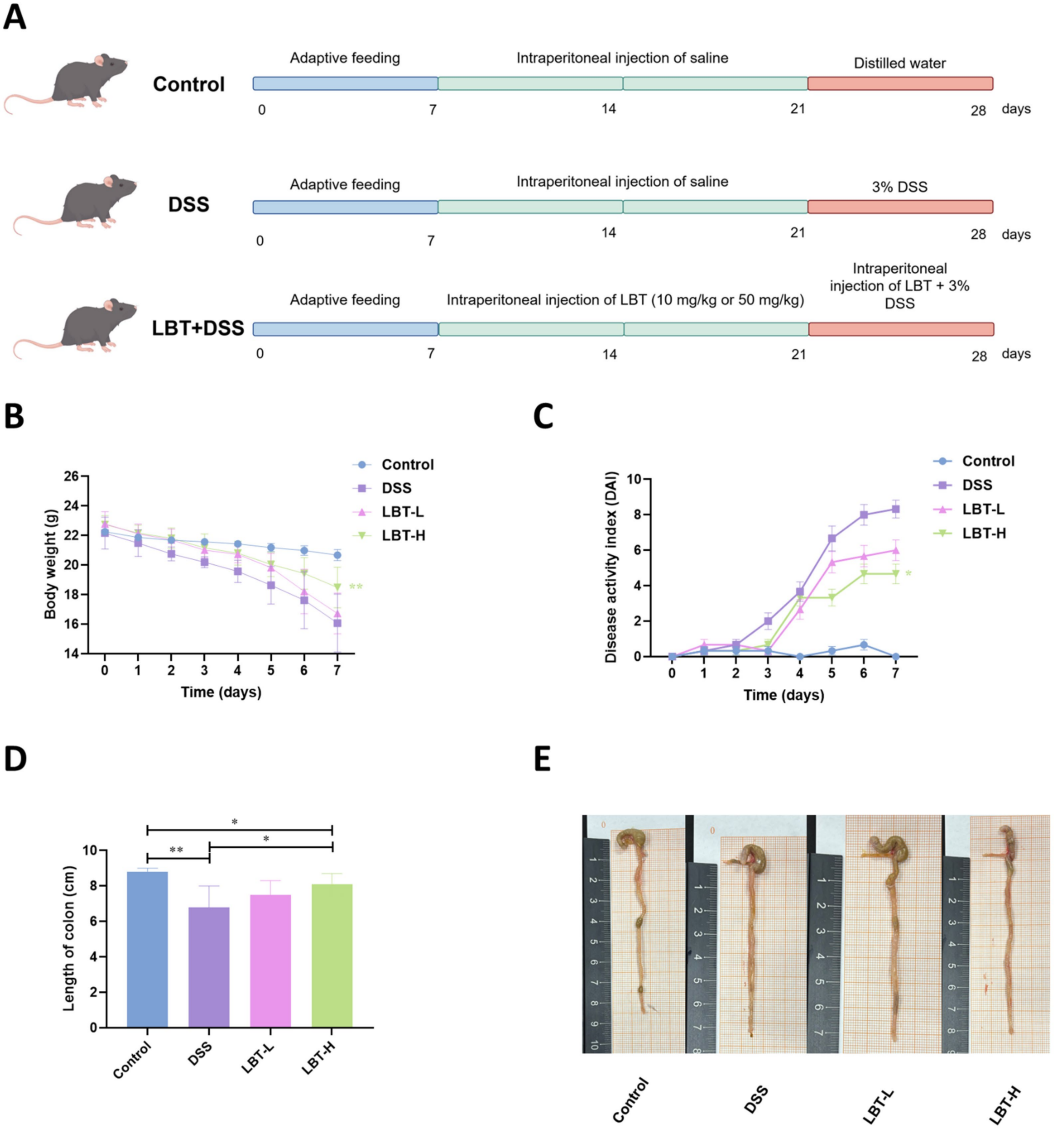
LBT inhibits DSS-induced colitis symptoms. **(A)** Flowchart of the experimental design. **(A)** Flowchart of the experimental design. **(B)** Body weight change curve. **(C)** DAI score curve. **(D)** Colon length and representative images **(E)** showing the length of the colon in each group of mice (*n* = 8). ^*^p < 0.05, ^**^p < 0.01.

A total of 32 male mice were randomly divided into four groups (*n* = 8 per group): Control, DSS (3% w/v), DSS + LBT-L (10 mg/kg), and DSS + LBT-H (50 mg/kg). The dosages of LBT were determined according to previous studies (Duan et al., 2025). Drug administration in each group was performed following the method described by Tang et al. (2024). The detailed treatment protocol and experimental procedure are illustrated in Figure 1A. During the experimental period, all mice were monitored daily to evaluate the disease activity index (DAI) score. On day 29, after overnight fasting, mice were anesthetized with isoflurane and euthanized by cervical dislocation. Blood samples were collected and centrifuged at 3000 rpm for 10 min, and the supernatants were stored at −80 °*C. colon* tissues were collected, with portions fixed in 4% paraformaldehyde for histological analysis, while the remaining samples were divided into three parts and stored at −80 °C for further assays. Colonic contents were snap-frozen in liquid nitrogen for 1 h and then stored at −80 °C until analysis.

### 2.3 Evaluation of disease activity index

DAI (Tang et al., 2024) was determined as the average of weight loss, stool consistency, and rectal bleeding scores:


$$\mathrm{DAI}=(\begin{array}{l} \text{Weight Loss Score}+\text{Stool Consistency Score}\\ +\text{Rectal Bleeding Score} \end{array})/3$$

The scoring criteria are shown in Table 1.

**Table 1:** DAI scoring criteria.

Parameter	Score	Criteria
Weight loss (%)	0	None
	1	1–5%
	2	5–10%
	3	10–20%
	4	>20%
Stool consistency	0	Normal
	1	Soft
	2	Loose
	3	Watery
Rectal bleeding	0	None
	1	Occult
	2	Gross
	3	Severe

Scoring criteria: weight loss, 0 (none), 1 (1–5%), 2 (5–10%), 3 (10–20%), 4 (>20%); stool consistency, 0 (normal), 1 (soft), 2 (loose), 3 (watery); rectal bleeding, 0 (none), 1 (occult), 2 (gross), 3 (severe).

### 2.4 Biochemical analysis

At first, mice were sacrificed, and blood samples were collected from the retro-orbital venous plexus. The blood samples were centrifuged at 3500 × g for 10 min at 4 °C to obtain the serum, and the corresponding serum biochemical indices were determined using commercial assay kits. In addition, mouse colon tissues were harvested and homogenized. The tissues were prepared as a 10% (w/v) homogenate in saline under ice-bath conditions, followed by centrifugation at 2500 × g for 10 min at 4 °C to collect the supernatant. The biochemical parameters of the colon homogenates were then measured using the appropriate kits. All procedures were conducted in strict accordance with the manufacturer’s protocols.

### 2.5 Histopathological staining

Colon tissues were fixed in 4% paraformaldehyde, embedded in paraffin, and sectioned at a thickness of 5 μm. The sections were subjected to haematoxylin-eosin (HE), alcian blue (AB), and periodic acid-Schiff (PAS) staining following standard protocols. HE staining was applied to evaluate histological structure and pathological lesions, while AB and PAS staining were performed to visualize goblet cell-secreted mucins and glycoproteins.

### 2.6 Determination of SCFAs

SCFAs in mouse intestinal contents were determined by GC–MS (GC-2030/QP2020 NX, Shimadzu, Japan) equipped with an HP-FFAP capillary column (30 m × 0.25 mm × 0.25 μm; Agilent, USA). Briefly, samples were homogenized in ultrapure water, sonicated, and centrifuged. The supernatant was mixed with 50% H₂SO₄ and methyl tert-butyl ether containing 2-methylvaleric acid (internal standard), vortexed, sonicated, and re-centrifuged. The resulting supernatant was subjected to GC–MS analysis under the following conditions: injection volume 1 μL (split 5:1), helium carrier gas 1.2 mL/min, injector temperature 220 °C, and oven program from 50 °C to 240 °C with multiple ramps. The transfer line, ion source, and quadrupole temperatures were 240 °C, 240 °C, and 150 °C, respectively, with electron impact ionization at −70 eV. Quantification was performed using external calibration curves of SCFA standards with 2-methylvaleric acid as the internal standard (*R*^2^ > 0.99).

### 2.7 16S rRNA gene sequencing and analysis

Genomic DNA was extracted from fecal samples using the OMEGA Soil DNA Kit (Omega Bio-Tek, USA). The V3-V4 regions of the bacterial 16S rRNA gene were amplified with primers 338F and 806R, and PCR products were purified using the Agencourt AMPure XP kit (Beckman Coulter, USA). Purified amplicons were quantified, pooled in equimolar amounts, and sequenced on an Illumina NovaSeq 6,000 platform (2 × 250 bp) by Shanghai Biotree Biotech Co., Ltd.

Raw reads were processed using Cutadapt (v 4.9) and DADA2 for quality filtering, denoising, merging, and chimera removal to obtain amplicon sequence variants (ASVs). Taxonomic classification was performed in QIIME2 (v 2024.2) against the SILVA 138 database (confidence ≥ 0.8). Alpha diversity (Chao1, Shannon, Simpson, Faith’s PD, and Pielou’s evenness) and beta diversity (Bray-Curtis, unweighted and weighted UniFrac) were calculated in QIIME2. Principal coordinate analysis (PCoA) and non-metric multidimensional scaling (NMDS) were performed in R (v 4.2.3). Differential taxa were identified using LEfSe with an LDA score > 3.0 and *p* < 0.05.

### 2.8 Western blotting and immunohistochemistry

Colon tissues were homogenized in ice-cold RIPA lysis buffer supplemented with protease and phosphatase inhibitors, followed by incubation on ice for 20 min. The homogenates were centrifuged at 12,000 *g* for 15 min at 4 °C to collect the supernatants containing total proteins. Protein concentrations were determined using the BCA protein assay kit. Equal amounts of protein were mixed with loading buffer, denatured at 95 °C for 5 min, separated by SDS-PAGE, and transferred onto PVDF membranes. After methanol activation, membranes were blocked with 5% non-fat milk in TBST for 2 h at room temperature and then incubated overnight at 4 °C with the indicated primary antibodies. After three washes in TBST (10 min each), membranes were incubated with HRP-conjugated secondary antibodies for 2 h at room temperature. Protein bands were detected using enhanced chemiluminescence (ECL) reagents and imaged with a Bio-Rad ChemDoc imaging system. Band intensities were quantified by ImageJ software. Immunohistochemistry was performed to assess Occludin expression in colon tissues. Paraffin-embedded sections (4 μm) were deparaffinized, rehydrated, and subjected to antigen retrieval in citrate buffer. After blocking endogenous peroxidase activity and nonspecific binding, the sections were incubated overnight at 4 °C with an anti-Occludin antibody (1:200) and subsequently processed using a commercial immunohistochemistry kit.

### 2.9 Nontargeted metabolomics

Approximately 25 ± 1 mg of frozen colonic content was accurately weighed into a 2 mL EP tube, followed by the addition of homogenization beads and 500 μL of extraction solvent (methanol: acetonitrile: water = 2:2:1, v/v/v) containing isotope-labeled internal standards. The mixture was vortexed for 30 s, homogenized at 35 Hz for 4 min, and sonicated in an ice-water bath for 5 min. This homogenization-sonication cycle was repeated three times to ensure complete extraction of metabolites. The samples were then incubated at −40 °C for 1 h to precipitate proteins and centrifuged at 12,000 rpm for 15 min at 4 °C. The supernatant was filtered through a 0.22 μm protein precipitation plate under positive pressure, and the filtrates were collected for analysis. Quality control samples were prepared by pooling equal aliquots from each supernatant.

Metabolomic profiling was performed on a Vanquish UHPLC system coupled with an Orbitrap Exploris 120 mass spectrometer (Thermo Fisher Scientific, USA). Polar metabolites were separated on a Waters ACQUITY UPLC BEH Amide column (2.1 × 50 mm, 1.7 μm). The mobile phases consisted of (A) water with 25 mmol/L ammonium acetate and 25 mmol/L ammonium hydroxide, and (B) acetonitrile. The column temperature was maintained at 4 °C, and the injection volume was 2 μL. Mass spectrometry was conducted in both positive and negative ion modes under the following parameters: sheath gas flow rate, 50 Arb; auxiliary gas flow rate, 15 Arb; capillary temperature, 320 °C; full MS resolution, 60,000; MS/MS resolution, 15,000; stepped collision energy, 20/30/40; spray voltage, 3.8 kV (positive) or −3.4 kV (negative).

Raw data were converted to mzXML format using ProteoWizard (v 3.0.24054) and processed with in-house R scripts. Metabolite identification was performed against the BiotreeDB database (v 3.0) following the Metabolomics Standards Initiative levels. Multivariate statistical analyses, including principal component analysis (PCA) was conducted using SIMCA (v 18.0.1, Sartorius Stedim Data Analytics AB, Umeå, Sweden).

### 2.10 Statistical analysis

All data are expressed as means ± SEM. Statistical analyses were conducted using SPSS software (version 22.0; IBM Corp., Armonk, NY, USA). Statistical differences between the Control group and the DSS or LBT treatment groups were analyzed using the Student’s *t*-test. Comparisons among multiple groups (DSS, LBT-L, and LBT-H) were performed using one-way analysis of variance (ANOVA), followed by Dunnett’s *post hoc* test to evaluate differences between LBT-treated groups and the DSS group. A value of *p* < 0.05 was considered statistically significant.

## 3 Results

### 3.1 LBT alleviates DSS-induced colitis symptoms in mice

To assess the ameliorative effect of LBT on DSS-induced colitis in mice, the present study firstly monitored the body weight changes, DAI scores, and colon length of mice in each group. The results showed that compared with the control group, mice in the DSS group showed a significant decrease in body weight from the 3rd day of administration, accompanied by a significant increase in the DAI score (Figures 1B,C). Both the low-dose group (LBT-L) and high-dose group (LBT-H) of LBT were able to attenuate the trend of body weight loss to varying degrees, with a more pronounced improvement in the LBT-H group (*p* < 0.01). Meanwhile, the length of the colon was significantly shortened in the DSS group (*p* < 0.01), whereas the LBT treatment significantly lengthened the length of the colon (LBT-H: *p* < 0.05) (Figures 1D,E). However, when comparing the LBT-H groups with the control group, the colon length in the LBT-H treatment group was significantly shorter than that in the control group (*p* < 0.05). These results indicated that LBT significantly alleviated the clinical symptoms of DSS-induced colitis in mice, reduced weight loss, decreased disease severity, and improved colon shortening.

### 3.2 LBT attenuates DSS-induced colitis inflammatory response and oxidative stress

**Figure 2:**
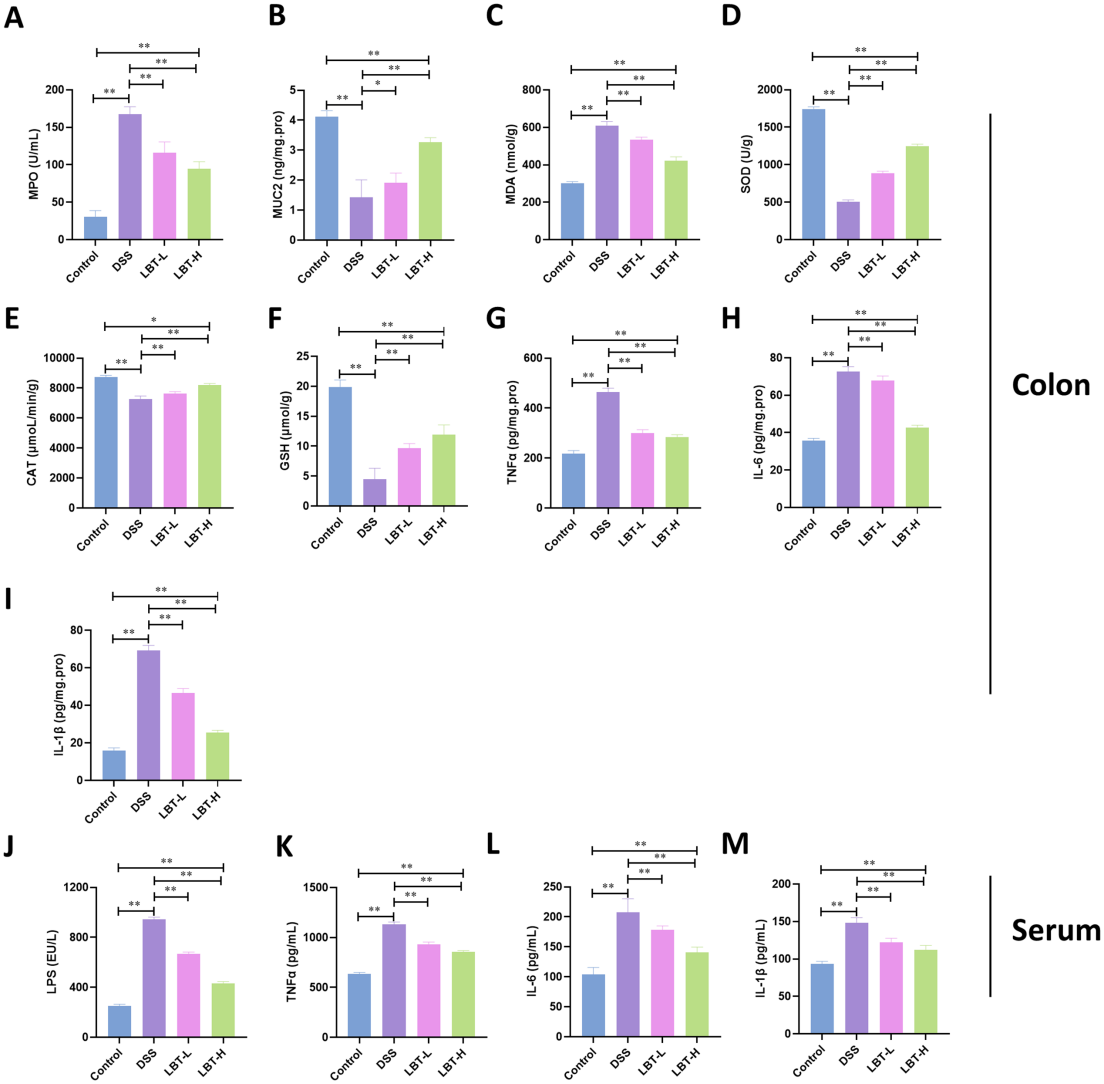
LBT inhibits DSS-induced inflammation and oxidative stress. **(A–I)** Effects of LBT on MPO, MUC2, MDA, SOD, CAT, GSH, TNFα, IL-6, and IL-1β in colon tissues. **(J–M)** Effect of LBT on LPS, TNFα, IL-6 and IL-1β in serum (*n* = 8). ^*^p < 0.05, ^**^p < 0.01.

To further explore the underlying mechanisms of LBT, we measured the changes in inflammatory cytokines and oxidative stress-related parameters in colon tissue and serum. Compared with the control group, mice in the DSS group exhibited a significant increase in myeloperoxidase (MPO) activity (*p* < 0.01) and a marked reduction in the goblet cell secretion marker MUC2 (*p* < 0.01), accompanied by elevated MDA levels (*p* < 0.01) and decreased levels of SOD, CAT, and reduced GSH (*p* < 0.01), indicating pronounced oxidative stress. LBT treatment significantly decreased MPO and MDA levels while increasing MUC2, SOD, CAT, and GSH levels, with more pronounced effects observed in the high-dose group (*p* < 0.05) (Figures 2A–F).

Regarding inflammatory cytokines, the levels of TNFα, IL-6, and IL-1β in colon tissue were significantly elevated in the DSS group (*p* < 0.01), along with increased serum concentrations of LPS, TNFα, IL-6, and IL-1β (*p* < 0.01). LBT treatment markedly reduced the levels of these inflammatory mediators in both colon tissue and serum, with the high-dose group exhibiting greater inhibitory effects (*p* < 0.05) (Figures 2G–M).

These findings indicate that LBT exerts protective effects against DSS-induced colitis by suppressing inflammatory responses, alleviating oxidative stress, and restoring mucus barrier components.

### 3.3 LBT improves intestinal barrier dysfunction induced by DSS

**Figure 3:**
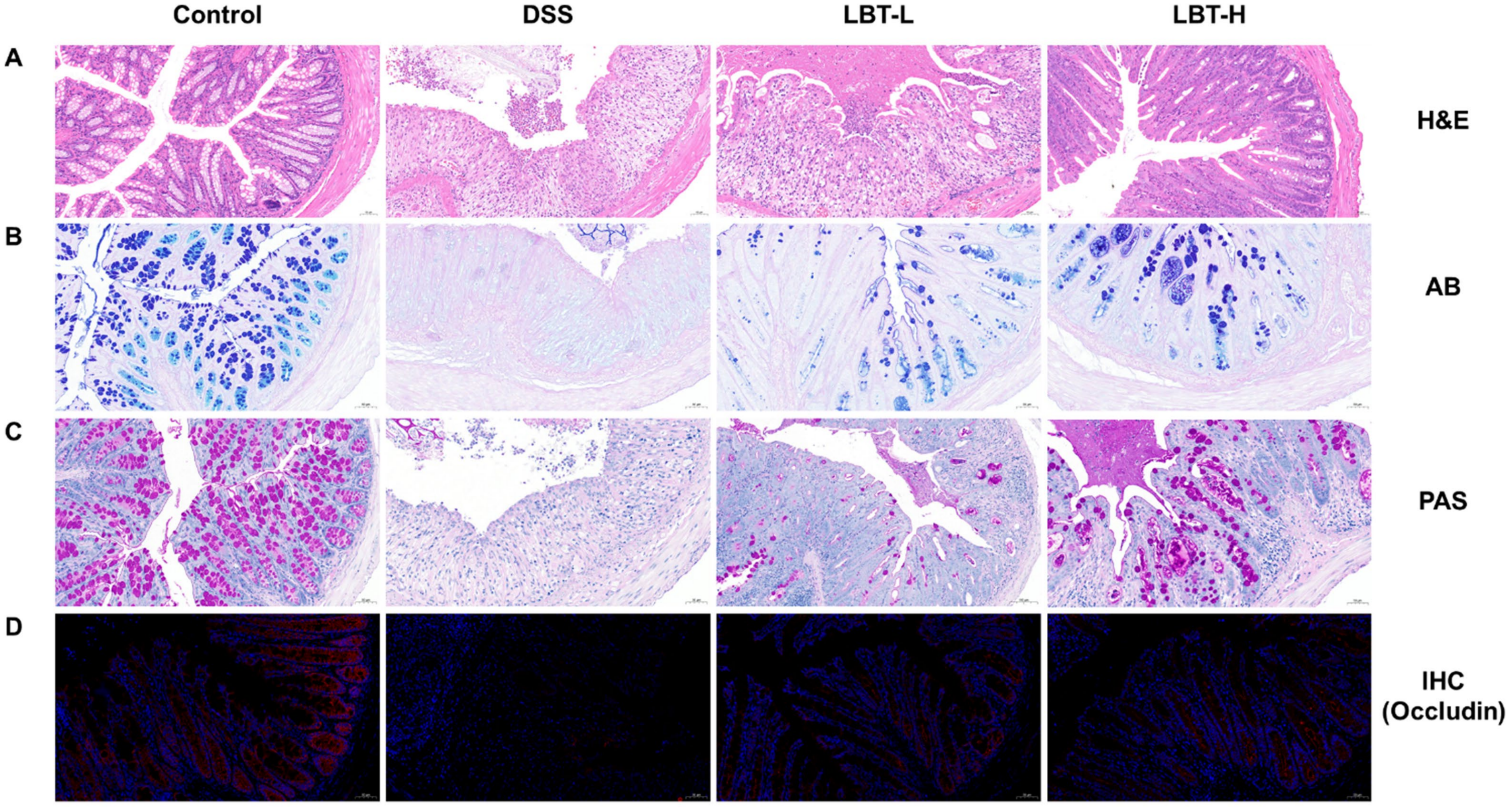
LBT attenuates DSS-induced intestinal barrier damage. **(A)** HE staining of colon tissue sections. **(B)** AB staining of colon tissue sections. **(C)** PAS staining of colon tissue sections. **(D)** Immunohistochemical detection of Occludin protein expression in colon tissue sections.

To evaluate the protective effect of LBT on intestinal barrier function, histopathological and histological analyses of colon tissues were performed. H&E staining showed that the control group exhibited intact colonic architecture with well-organized crypts, whereas the DSS group displayed disrupted crypt structures, epithelial cell shedding, and extensive inflammatory cell infiltration. LBT treatment markedly ameliorated these pathological damages, with the high-dose group (LBT-H) showing more pronounced protective effects (Figure 3A). Moreover, AB and PAS staining revealed that goblet cell numbers and mucus secretion were significantly reduced in the DSS group, while LBT intervention restored mucus layer distribution and integrity (Figures 3B,C).

**Figure 4:**
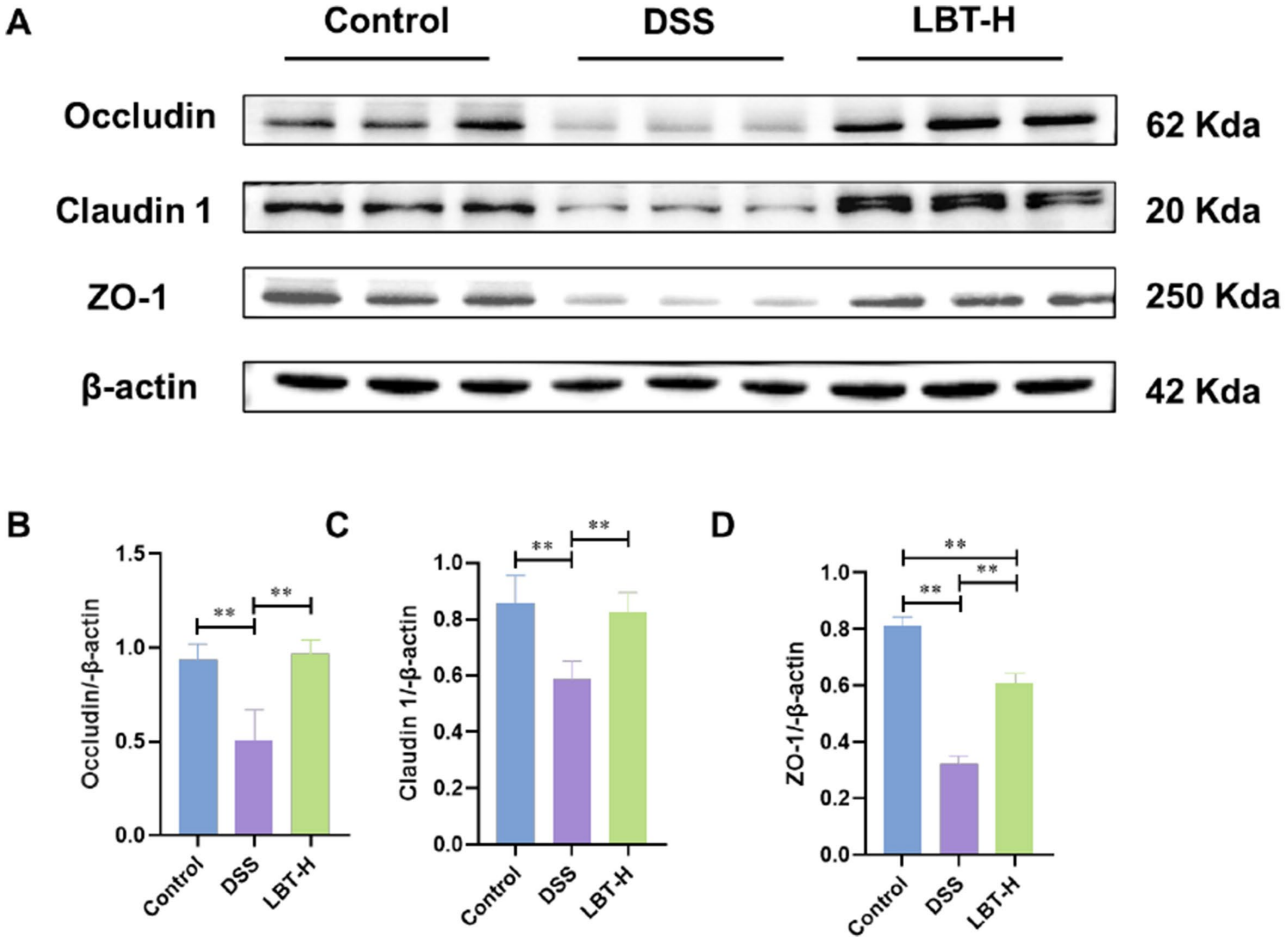
Effects of LBT on the expression of tight junction proteins. **(A)** Representative Western blot analysis of tight junction proteins (Occludin, Claudin-1, and ZO-1). **(B–D)** Quantitative analysis of Occludin, Claudin-1, and ZO-1 protein expression levels (*n* = 8). ^**^p < 0.01.

At the protein level, immunohistochemical staining demonstrated that DSS markedly downregulated the expression of the tight junction protein Occludin in colonic epithelium, whereas LBT administration effectively restored its expression (Figure 3D). Western blot analysis further showed that the protein levels of Occludin, Claudin-1, and ZO-1 were significantly decreased in the DSS group compared with the control group (*p* < 0.01) (Figures 4A–D). LBT treatment, particularly at the high dose, significantly upregulated the expression of these tight junction proteins (*p* < 0.01).

Collectively, these findings indicate that LBT alleviates DSS-induced intestinal barrier dysfunction by restoring goblet cell function and enhancing the expression of tight junction proteins, thereby exerting protective effects.

### 3.4 LBT improves SCFAs levels in DSS-induced colitis

**Figure 5:**
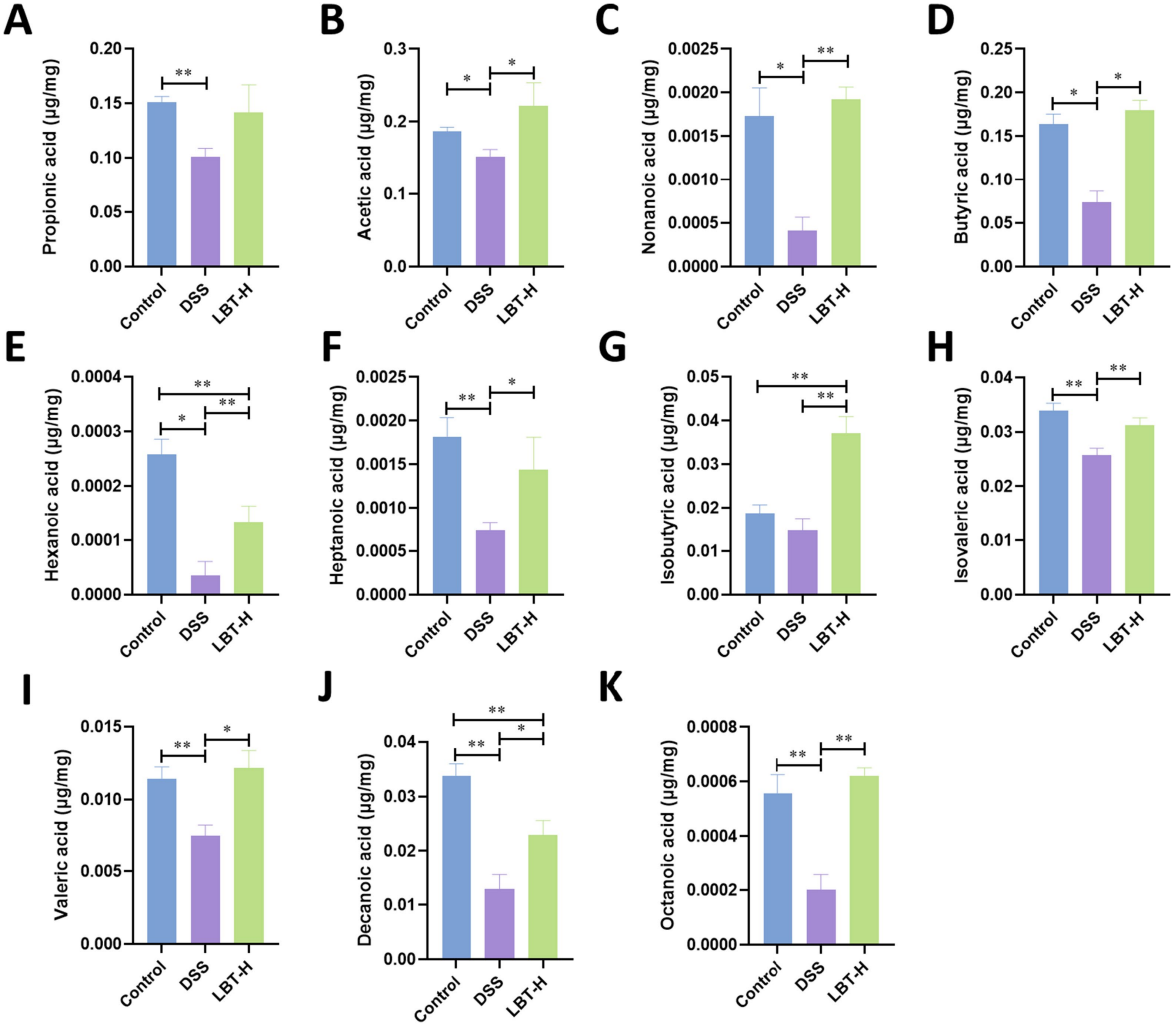
LBT improves DSS-induced intestinal short-chain fatty acid levels. **(A–K)** Effect of LBT on intestinal levels of Propionic acid, Acetic acid, Nonanoic acid, Butyric acid, Hexanoic acid, Heptanoic acid, Isobutyric acid, Isovaleric acid, Valeric acid, Decanoic acid and Octanoic acid (*n* = 8). ^*^p < 0.05, ^**^p < 0.01.

To further investigate the regulatory effects of LBT on intestinal metabolites, we measured the levels of various SCFAs in colonic contents. Compared with the control group, mice in the DSS group exhibited significantly reduced levels of multiple SCFAs, including propionic acid, acetic acid, nonanoic acid, butyric acid, hexanoic acid, heptanoic acid, isovaleric acid, valeric acid, decanoic acid, and octanoic acid (*p* < 0.05), indicating a marked disruption of SCFA metabolism associated with DSS-induced colitis (Figure 5). Notably, LBT administration effectively restored the levels of these SCFAs toward control group, which are the SCFAs critical for maintaining intestinal barrier function and immune homeostasis. These results suggest that LBT ameliorates colonic metabolic disturbances by promoting SCFA recovery in DSS-induced colitis.

### 3.5 LBT ameliorates DSS-induced gut microbiota dysbiosis

**Figure 6:**
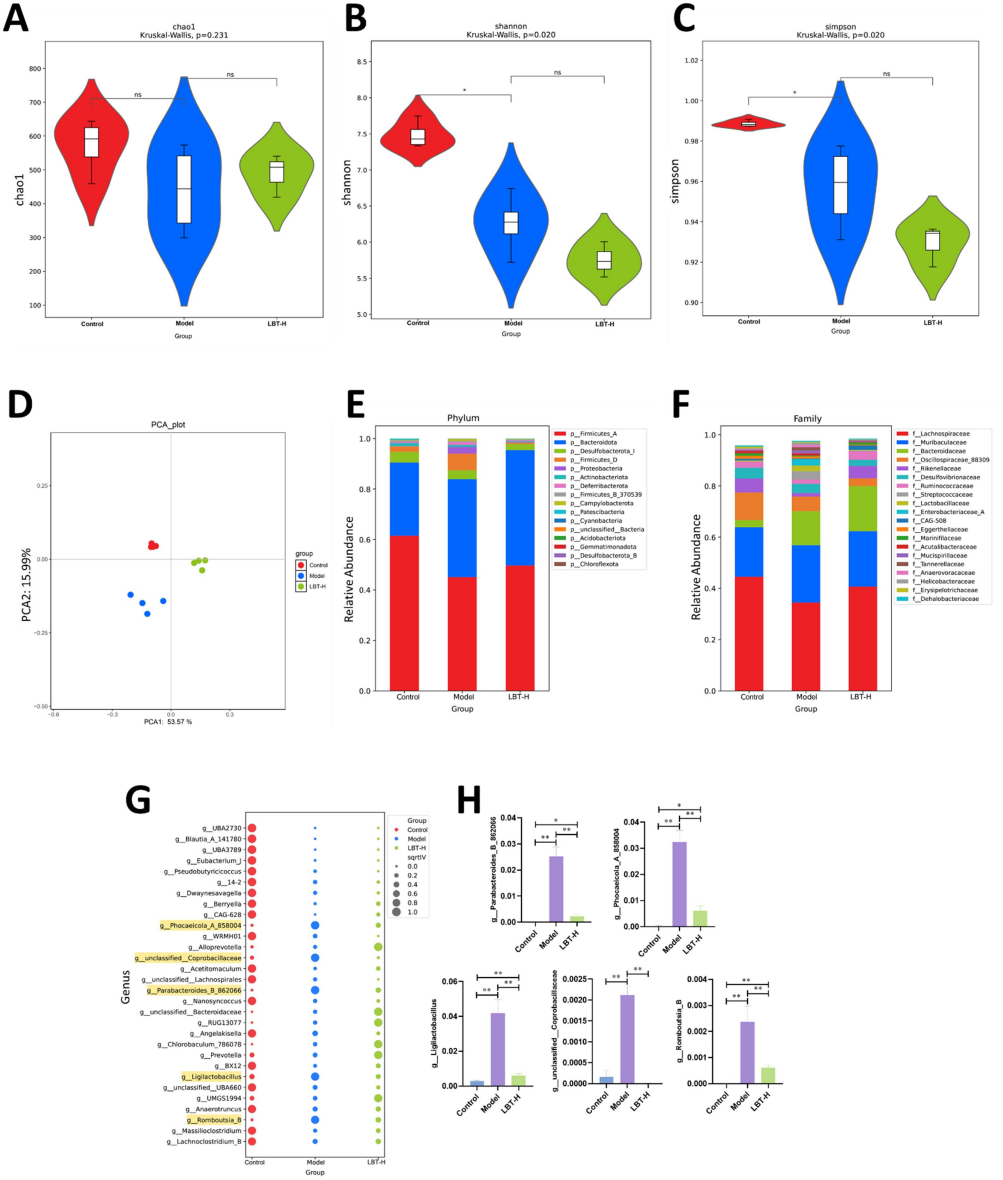
LBT attenuated DSS-induced gut microbiota dysbiosis. **(A)** Chao1 index; **(B)** Shannon index; **(C)** Simpson index; **(D)** PCA analysis. **(E)** Species composition plot at the phylum level. **(F)** Species composition plot at the family level. **(G)** Results of the indicative species analysis at the genus level. **(H)** Abundance of g_*Parabacteroides*_B_862066, g_*Phocaeicola*_A_858004, g_*Ligilactobacillus*, g_unclassified_*Coprobacillaceae*, and g _*Romboutsia*_B in each group was determined (*n* = 4). ^**^p < 0.01.

To investigate the regulatory effect of LBT on gut microbiota, 16S rRNA sequencing was performed on caecal contents from different groups. Alpha diversity analysis showed that the Shannon and Simpson indices were significantly reduced in the DSS model group compared with the control group (*p* < 0.05), indicating that DSS-induced colitis led to a decline in microbial diversity and evenness. In contrast, LBT intervention did not significantly restore these two indices. The Chao1 index showed no significant difference among the groups, suggesting that the overall species richness of the gut microbiota remained relatively stable despite changes in community structure (Figures 6A–C). *β* diversity was assessed using principal component analysis (PCA), which demonstrated clear separation of microbial community structures among the three groups. The DSS group exhibited a marked difference from the control group, while the microbial community of the LBT group showed a trend of shifting back towards that of the control group (Figure 6D).

At the phylum level, Firmicutes and Bacteroidota were dominant across all groups. Compared with the control group, the relative abundance of Firmicutes was notably decreased in the DSS group, whereas LBT treatment partially reversed this alteration (Figure 6E). At the family level, DSS treatment led to an increased abundance of Bacteroidaceae and a reduction in Lachnospiraceae. Importantly, LBT intervention restored the decreased abundance of Lachnospiraceae and alleviated the microbial imbalance (Figure 6F).

Genus-level analysis revealed that LBT treatment effectively modulated the abundance of several genera (Figure 6G). Further quantitative analysis demonstrated that LBT significantly reversed the DSS-induced increase in g_*Parabacteroides*_B_862066, g_*Phocaeicola*_A_858004, g_*Ligilactobacillus*, g_unclassified_*Coprobacillaceae*, and g_*Romboutsia*_B (*p* < 0.01, Figure 6H). Collectively, these results suggest that LBT effectively improves DSS-induced gut microbiota dysbiosis by restoring microbial diversity and community composition.

### 3.6 Correlation between LBT-regulated gut microbiota and SCFAs

**Figure 7:**
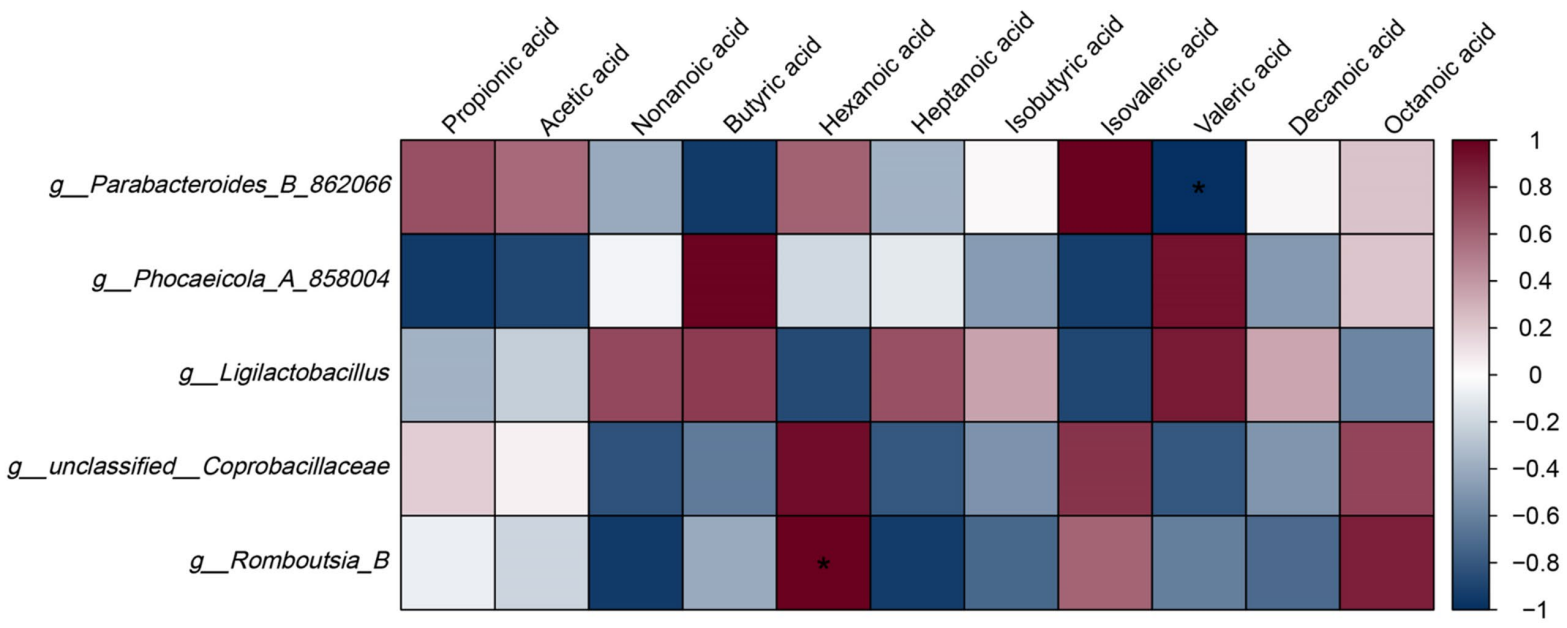
Correlation between LBT-regulated gut microbiota and SCFAs (*n* = 3). ^**^p < 0.01.

To elucidate the link between LBT-mediated microbial shifts and metabolic outputs, we analyzed correlations between the relative abundances of key genera and SCFA levels in caecal contents (Figure 7). The heatmap revealed distinct association patterns across taxa and metabolites. Notably, *g_Parabacteroides_B_862066* showed a significant negative correlation with valeric acid (*p* < 0.05), whereas *g__Romboutsia_B* exhibited a significant positive correlation with hexanoic acid (*p* < 0.05). In addition, *g__Phocaeicola_A_858004*, *g__Ligilactobacillus*, and *g__unclassified__Coprobacillaceae* displayed varying degrees of positive or negative correlations with multiple SCFAs, indicating complex interactions between community composition and SCFA profiles. Collectively, these results suggest that the microbiota changes elicited by LBT are closely associated with alterations in SCFA patterns, supporting a potential microbiota-metabolite axis underlying the observed benefits in DSS-induced colitis.

### 3.7 Regulatory effect of LBT on DSS-induced intestinal metabolic alterations

**Figure 8:**
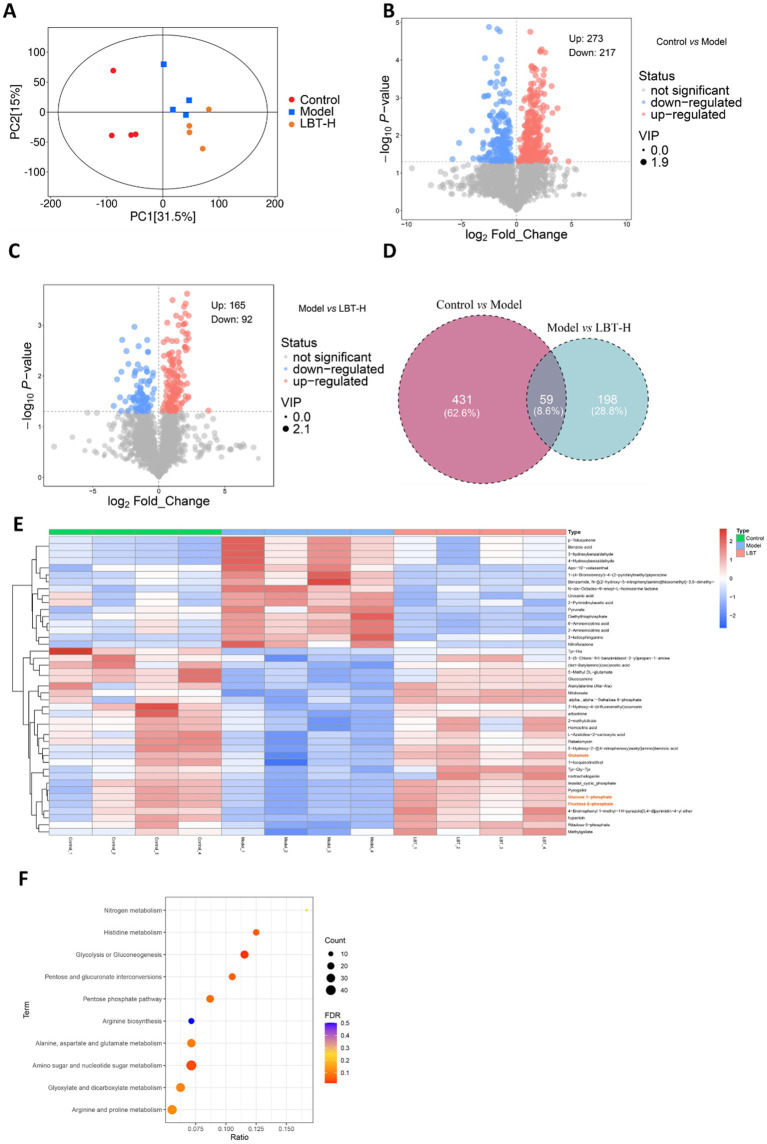
LBT alleviates DSS-induced intestinal metabolic disorders. **(A)** PCA analysis of metabolites of colonic contents in different groups. **(B)** Volcano plot of metabolites in Model group compared to Control group. **(C)** Volcano plot of metabolites in LBT group compared to the Model group. **(D)** Venn diagram showing differential metabolites between the three groups. **(E)** Heat map of differential metabolites regulated by LBT. **(F)** KEGG enrichment analysis of differential metabolites regulated by LBT.

To investigate the regulatory effect of LBT on DSS-induced intestinal metabolic disorders, nontargeted metabolomics analysis was performed on colonic contents from different groups of mice. PCA analysis revealed a clear separation of metabolic profiles among the Control, Model, and LBT-H groups, indicating that LBT intervention markedly altered the metabolic composition disturbed by DSS (Figure 8A). Volcano plot analysis showed that, compared with the Control group, the Model group exhibited 273 upregulated and 217 downregulated metabolites (Figure 8B). In contrast, compared with the Model group, the LBT-H group displayed 165 significantly upregulated and 92 significantly downregulated metabolites (Figure 8C). Venn diagram analysis further demonstrated that 59 differential metabolites overlapped between the two comparisons (Figure 8D), suggesting that these metabolites may be specifically modulated by LBT.

Heatmap-based hierarchical clustering of these differential metabolites indicated that LBT intervention effectively reversed the DSS-induced metabolic alterations (Figure 8E). KEGG enrichment analysis of these metabolites revealed that the most significantly affected pathway was amino sugar and nucleotide sugar metabolism (Figure 8F). Moreover, heatmap analysis highlighted that LBT restored the abundance of key metabolites within this pathway, including glutamate, glucose 1-phosphate, and fructose 6-phosphate (Figure 8E). These findings suggest that LBT may exert its protective effect against DSS-induced intestinal metabolic disorders primarily through the modulation of Amino sugar and nucleotide sugar metabolism.

## 4 Discussion

In this study, we demonstrated that LBT, an active component derived from traditional Chinese medicine, significantly ameliorates DSS-induced colonic injury via multifaceted mechanisms including suppression of inflammation and oxidative stress, reinforcement of epithelial barrier integrity, modulation of gut microbiota, and metabolic reprogramming-particularly targeting the amino sugar and nucleotide sugar metabolism.

DSS-induced colitis is characterized by elevated MPO activity, pro-inflammatory cytokines (TNF-*α*, IL-6, IL-1β), and lipid peroxidation, alongside depleted antioxidant defenses (SOD, CAT, GSH). LBT administration reversed these biochemical derangements, especially at the higher dose, indicating that LBT may neutralize neutrophil infiltration and excessive ROS production. Consistent with other traditional Chinese medicine (TCM)-derived bioactives, such as *Allium sativum* L. extract and *Astragalus* polysaccharide, LBT exerts a dual anti-inflammatory and antioxidant effect through modulation of classical signaling cascades (NF-κB, COX-2, and TNF-α). *Allium sativum* L. extracts are rich in polyphenols, with catechins as the main component, which significantly reduce colonic inflammation and oxidative damage through potent inhibition of COX-2, TNF-α, NF-κB, IL-6, and PGE₂ and reduction of 8-iso-PGF₂α (Recinella et al., 2022). Astragalus polysaccharide significantly alleviates colonic damage in ulcerative colitis by inhibiting pro-inflammatory factors (TNF-α, IL-6, IL-1β) and oxidative stress products (MDA) and restoring the activity of antioxidant enzymes (SOD) (Hu et al., 2022). Likewise, *A. macrocephala*-derived carbon dots (AM-CDs) effectively eliminate hydroxyl (·OH) and superoxide (·O₂-) radicals and modulate PI3K-Akt/MAPK and Jak–STAT/MAPK pathways (Li et al., 2025). Therefore, the anti-inflammatory and antioxidant properties of LBT may share mechanistic similarities with these traditional medicinal compounds, while exhibiting a broader and more integrated bioactivity spectrum.

The integrity of the intestinal barrier is essential in preventing luminal antigen translocation and sustaining mucosal homeostasis. Histological and immunohistochemical assessments in this study revealed that LBT rescued goblet cell depletion, mucus layer disruption, and crypt architecture distortion, while upregulating tight junction proteins (Occludin, Claudin-1, ZO-1). In UC, goblet cells and mucins are central to maintaining intestinal mucus integrity (Liu et al., 2024), while tight junction proteins maintain epithelial cohesion and barrier function (Qu et al., 2024). Compared with other TCM-derived barrier-protective agents, such as Biochanin A and Baitouweng Decoction, LBT demonstrated a more comprehensive mucosal-restorative effect. Biochanin A repaired intestinal barrier function by up-regulating ZO-1, Occludin, and Claudin-1 and restoring goblet cells (Zhang et al., 2024). Baitouweng Decoction alleviated DSS-induced colitis by enhancing tight junction proteins and inhibiting bacterial translocation (Pan et al., 2024). In contrast, LBT not only upregulates tight junction proteins and restores goblet cell numbers but also maintains crypt architecture and mucus layer continuity, suggesting that it acts as a multi-target modulator within the TCM framework to reinforce intestinal barrier integrity.

Lobetyolin treatment significantly increased SCFA levels while reducing the relative abundances of *g_Parabacteroides_B_862066, g_Phocaeicola_A_858004, g_Ligilactobacillus, g_unclassified_Coprobacillaceae,* and *g_Romboutsia_B*. These microbial shifts suggest that LBT alleviates intestinal inflammation partly through rebalancing the gut microbiota and restoring microbial metabolic function. SCFAs such as acetate, propionate, and butyrate are key metabolites that fuel colonocytes and exert anti-inflammatory and barrier-protective effects (Yue et al., 2022). The decreased abundance of *Parabacteroides* and *Phocaeicola*, which are associated with LPS production and pro-inflammatory signaling, may contribute to the attenuation of mucosal inflammation (Ling et al., 2022; Huang et al., 2025b). Similarly, reductions in *Coprobacillaceae* and *Romboutsia*, often linked to dysbiosis and epithelial barrier disruption, indicate restoration of intestinal homeostasis (Jia et al., 2023; Zeng et al., 2025). The concurrent elevation of SCFAs further supports recovery of beneficial microbial metabolism. Comparable regulatory effects have been reported for other TCM-derived compounds, such as *Ganoderma lucidum* polysaccharides and berberine, which mitigate colitis by enhancing SCFA-producing bacteria and suppressing inflammatory taxa (Feng et al., 2022; Zhou et al., 2023). Thus, LBT may exert its therapeutic efficacy through coordinated modulation of gut microbiota composition and metabolic output, leading to suppression of inflammation and reinforcement of the mucosal barrier in UC.

The metabolomics analysis demonstrated that DSS-induced colitis caused profound disturbances in amino sugar and nucleotide sugar metabolism, which are crucial for mucin glycosylation, epithelial function, and immune regulation (Rao and Samak, 2011; Miner-Williams and Moughan, 2016). Alterations in glutamate, glucose 1-phosphate, and fructose 6-phosphate disrupt epithelial energy supply and glycosylation, aggravating mucosal injury (Dong et al., 2024). LBT restored these key metabolites, suggesting that it may regulate energy and glycan metabolism in a manner comparable to other TCM compounds such as ginsenosides and astragalosides, known to normalize carbohydrate metabolism and enhance epithelial resilience (Zhou et al., 2021; Han et al., 2025). Thus, LBT’s protective effects may be mediated by restoring amino sugar and nucleotide sugar metabolism, maintaining epithelial integrity, and attenuating inflammatory responses.

Overall, these findings support the concept that LBT represents a multifunctional TCM-derived agent with integrated anti-inflammatory, antioxidant, microbiota-modulating, and metabolic-regulatory properties. Its broad spectrum of actions and mechanistic overlap with other well-characterized traditional herbal compounds underscores its potential as a novel therapeutic candidate for ulcerative colitis. LBT has been reported to exert anti-inflammatory and neuroprotective effects by promoting the M1-to-M2 polarization of microglia and downregulating TNF-*α*, while simultaneously targeting Aβ aggregation, oxidative stress, and glutathione metabolism, with no apparent toxicity observed in *Caenorhabditis elegans* models (Huang et al., 2025a). Future studies should include mechanistic validation using antibiotic or germ-free models, targeted metabolomics with isotope tracing, and comprehensive PK/PD profiling to advance LBT toward clinical application within the TCM-based drug discovery paradigm.

## Data Availability

The data presented in the study are deposited in the figshare repository, accession number 10.6084/m9.figshare.30606413.
