# Supplementary figures and images for: Lobetyolin ameliorates DSS-induced ulcerative colitis in mice by alleviating inflammation, restoring barrier function, and modulating gut microbiota–metabolite interactions
Source: Front Microbiol. 2025 Nov 28;16:1710707. doi: 10.3389/fmicb.2025.1710707 (PMC12698648; doi:10.3389/fmicb.2025.1710707)

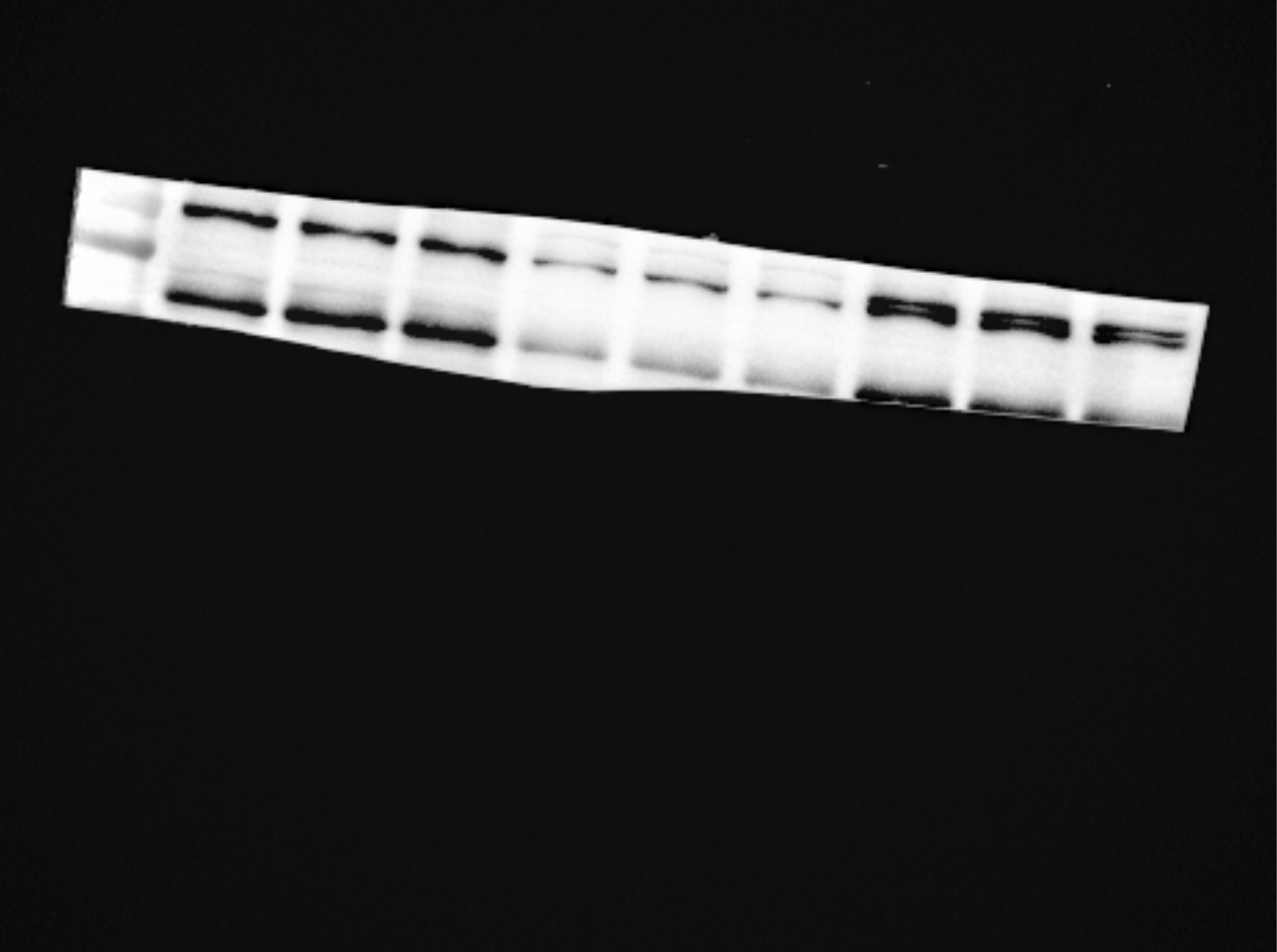

Supplement: Supplementary file 1 [file Image_1.jpeg]

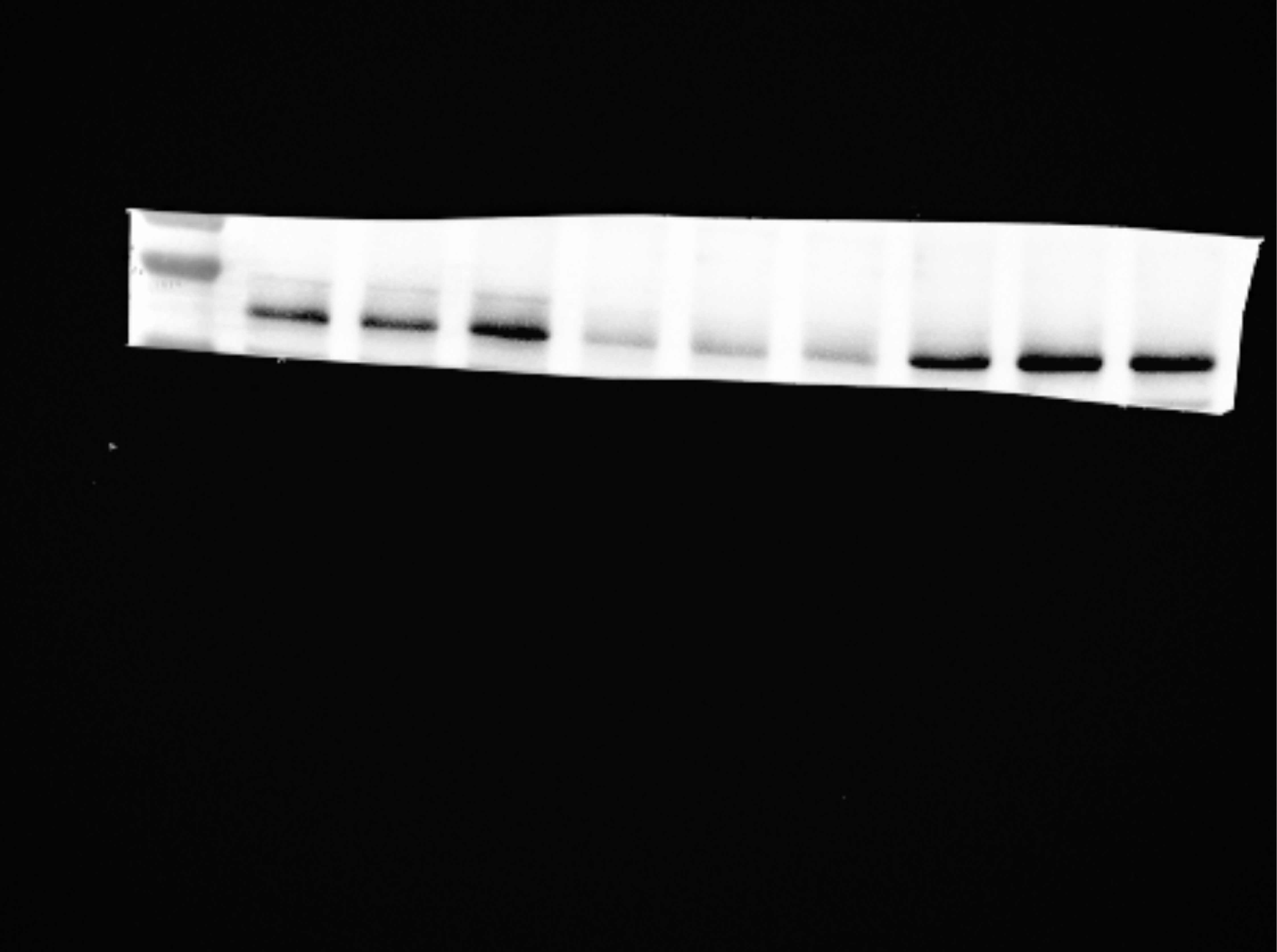

Supplement: Supplementary file 2 [file Image_2.jpeg]

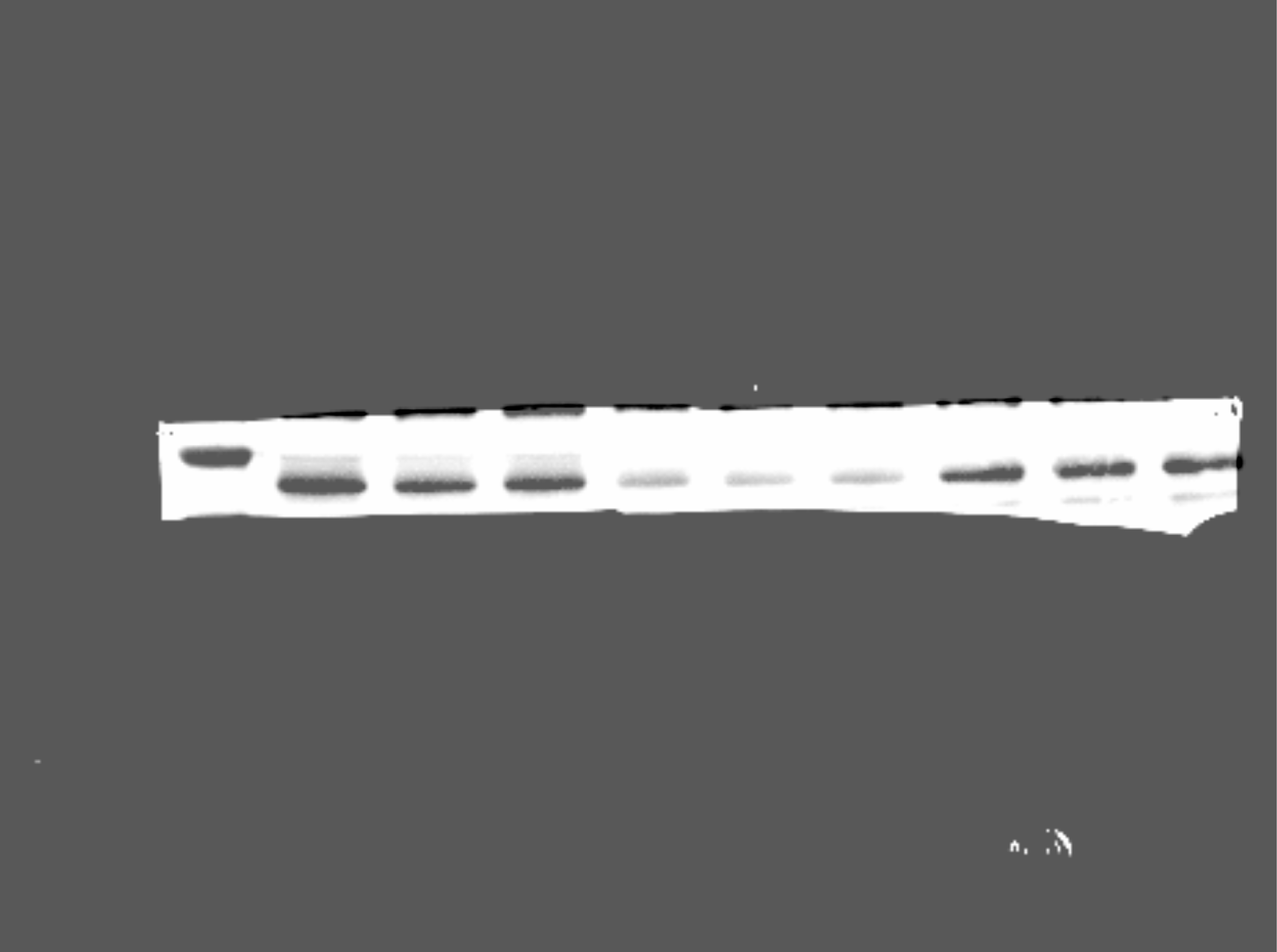

Supplement: Supplementary file 3 [file Image_3.jpeg]

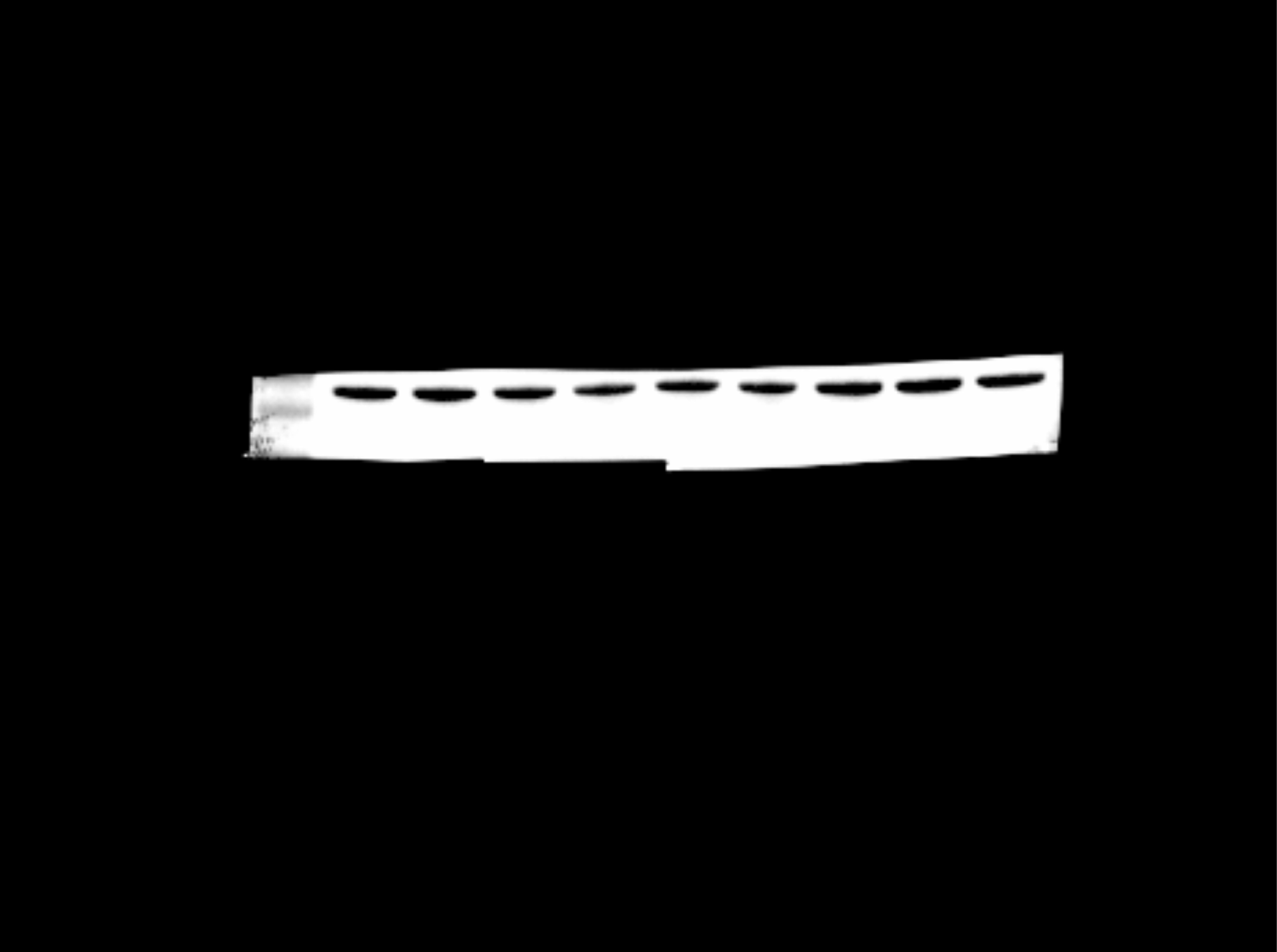

Supplement: Supplementary file 4 [file Image_4.jpeg]
